# Supplementary material for: A tree based eXtreme Gradient Boosting (XGBoost) machine learning model to forecast the annual rice production in Bangladesh
Source: PLoS One. 2023 Mar 27;18(3):e0283452. doi: 10.1371/journal.pone.0283452 (PMC10042373; doi:10.1371/journal.pone.0283452)
Supplement: S1 File — (DOCX) [file pone.0283452.s001.docx]

**A tree based eXtreme Gradient Boosting (XGBoost) machine learning model to forecast the Annual Rice Production in Bangladesh**

We developed an ARIMA and a XGBoost model in our study. We split our data set into two sets: Training and testing. The training data set includes 90% of the data and the remaining 10% as test set. To develop the ARIMA model in the training set, we have passed through different procedures. We found our data is not smooth. So, we first applied the parametric Box-Cox transformation to make our data set stable i.e., make the raw the smooth and exhibit less variation. The result after the Box-Cox transformation is shown in S1 Table.

**S1 Table. Comparison Box-Cox transformed sequence and the actual sequence of annual rice production data in Bangladesh**

| **Time** | **Actual** | **Box-Cox Transformed** |
| --- | --- | --- |
| 1961 | 14426184 | 114.23 |
| 1962 | 13304520 | 112.42 |
| 1963 | 15934944 | 116.50 |
| 1964 | 15753588 | 116.24 |
| 1965 | 15750540 | 116.24 |
| 1966 | 14363000 | 114.14 |
| 1967 | 16757000 | 117.67 |
| 1968 | 17015504 | 118.03 |
| 1969 | 18007296 | 119.35 |
| 1970 | 16714900 | 117.61 |
| 1971 | 14896689 | 114.96 |
| 1972 | 15133678 | 115.32 |
| 1973 | 17862704 | 119.16 |
| 1974 | 16929568 | 117.91 |
| 1975 | 19142624 | 120.80 |
| 1976 | 17628320 | 118.85 |
| 1977 | 19451488 | 121.19 |
| 1978 | 19582160 | 121.35 |
| 1979 | 19109472 | 120.76 |
| 1980 | 20821008 | 122.83 |
| 1981 | 20445872 | 122.39 |
| 1982 | 21324640 | 123.41 |
| 1983 | 21761008 | 123.90 |
| 1984 | 21933456 | 124.09 |
| 1985 | 22556288 | 124.78 |
| 1986 | 23110032 | 125.38 |
| 1987 | 23120624 | 125.39 |
| 1988 | 23316464 | 125.60 |
| 1989 | 26784480 | 129.08 |
| 1990 | 26777904 | 129.07 |
| 1991 | 27242000 | 129.51 |
| 1992 | 27373000 | 129.63 |
| 1993 | 26928000 | 129.21 |
| 1994 | 25124000 | 127.46 |
| 1995 | 26399000 | 128.71 |
| 1996 | 28182000 | 130.38 |
| 1997 | 28152000 | 130.35 |
| 1998 | 29710000 | 131.74 |
| 1999 | 34430000 | 135.61 |
| 2000 | 37627500 | 138.00 |
| 2001 | 36269000 | 137.01 |
| 2002 | 37593000 | 137.98 |
| 2003 | 38361420 | 138.52 |
| 2004 | 36235976 | 136.98 |
| 2005 | 39795616 | 139.53 |
| 2006 | 40773000 | 140.19 |
| 2007 | 43181000 | 141.78 |
| 2008 | 46742000 | 144.00 |
| 2009 | 48144000 | 144.84 |
| 2010 | 50061200 | 145.95 |
| 2011 | 50627000 | 146.27 |
| 2012 | 50497000 | 146.20 |
| 2013 | 51534000 | 146.78 |
| 2014 | 51806593 | 146.94 |

After that, we applied ADF test and found the data nonstationary. So, we performed first difference and made the data stationary. And also plot the ACF and PACF plot to find the value of p and q order. We used ‘auto.arima’ function to list all possible ARIMA model and then selected the ARIMA (0,1,1) with drift model as it contain lowest AICc value. The drift parameter indicates that our data drifts upward positively.

The parameters of the fitted ARIMA model were shown in the main text. The point estimates of the ARIMA model with the 95% confidence interval were shown in S2 Table.

**S2 Table. Point forecast with 95% confidence interval of ARIMA (0,1,1) with drift**

| **Time** | **Forecast** | **Lower CI** | **Upper CI** |
| --- | --- | --- | --- |
| 2015 | 53287781 | 47923001 | 59129527 |
| 2016 | 54456611 | 47923890 | 61693462 |
| 2017 | 55646143 | 48082072 | 64147585 |
| 2018 | 56856657 | 48344267 | 66545822 |
| 2019 | 58088435 | 48682730 | 68916567 |
| 2020 | 59341762 | 49080968 | 71276972 |

ARIMA: Autoregressive Integrated Moving Average

The XGBoost model in the training set was developed by frequently adjusting the different parameter. To build the XGBoost model of the rice production time series data, we used the ‘forecastxgb’ package. It allowed us to build the model easily. We randomly searched several parameters. We selected the trend_method as differencing because our data gets stationary after first differencing and seas_method as none because of having no seasonality. We found the best tuning parameters are nrounds = 8, nrounds_method = ‘cv’, nfold = 10, lambda = 0.1892, seas_method = ‘none’, and trend_method = ‘differencing’ as these give lowest MAPE value (S3 Table).

**S3 Table. Tunning parameters of the XGBoost model in the training set**

| **nrounds** | **nrounds_method** | **nfold** | **lambda** | **seas_method** | **trend_method** | **MAPE** |
| --- | --- | --- | --- | --- | --- | --- |
| 200 | cv | 10 | 0.3 | none | differencing | 19.75 |
| 200 | cv | 5 | 0.3 | none | differencing | 13.9 |
| 200 | cv | 5 | 0.2 | none | differencing | 10.41 |
| 100 | cv | 10 | 0.3 | none | differencing | 19.75 |
| 100 | cv | 5 | 0.3 | none | differencing | 19.75 |
| 100 | cv | 10 | 0.2 | none | differencing | 10.41 |
| 10 | cv | 10 | 0.3 | none | differencing | 13.9 |
| 10 | cv | 5 | 0.3 | none | differencing | 19.75 |
| 9 | cv | 10 | 0.1892 | none | differencing | 10.41 |
| 8 | cv | 10 | 0.1892 | none | differencing | 10.39 |

**S4 Table. Point forecast of XGBoost forecasting model**

| **Time** | **Forecast** |
| --- | --- |
| 2015 | 52780937 |
| 2016 | 53770086 |
| 2017 | 54774213 |
| 2018 | 55793490 |
| 2019 | 56828094 |
| 2020 | 57878199 |

XGBoost: eXtreme Gradient Boosting

We predicted the annual rice production in Bangladesh using our preferred XGBoost machine learning model (S5 Table).

**S5 Table. Point forecast of annual rice production in Bangladesh for next 10 years using XGBoost model**

| **Time** | **Forecast** |
| --- | --- |
| 2021 | 57850318 |
| 2022 | 61237213 |
| 2023 | 64444005 |
| 2024 | 66931730 |
| 2025 | 69917838 |
| 2026 | 71516432 |
| 2027 | 73543665 |
| 2028 | 76271911 |
| 2029 | 80422408 |
| 2030 | 82256940 |

XGBoost: eXtreme Gradient Boosting
