# Supplementary material for: A tree based eXtreme Gradient Boosting (XGBoost) machine learning model to forecast the annual rice production in Bangladesh
Source: PLoS One. 2023 Mar 27;18(3):e0283452. doi: 10.1371/journal.pone.0283452 (PMC10042373; doi:10.1371/journal.pone.0283452)
Supplement: Attachment — Submitted filename: Response to Reviewers.docx [file pone.0283452.s002.docx]

**PLOS ONE**

**PONE-D-22-20989**

**Accuracy Performance of Time Series and Machine Learning Models for Predicting Rice Production in Bangladesh: A Comparative Analysis**

We express gratitude to the reviewers and editors for their insightful remarks that helped us to enrich our paper. We have meticulously reworked each portion of the paper in response to the reviewers' and editors' feedback. We have also updated the title of the manuscript in accordance with the authors' decision. We have altered the entire manuscript significantly. We hope that the modifications made to the amended version (as shown below) will acceptable. All changes have been highlighted (yellow colour) with track changes in the revised manuscript.

**Reviewers’ Comments**

# COMMENTS TO AUTHORS’ SPECIFIC RESPONSES

| **Reviewer 1 Comments** | **Reviewers’ Responses to Questions** |
| --- | --- |
| 1. Line number is missing which made the review difficult. The language of the manuscript needs fine tuning. | 1. Thanks to the reviewers for his valuable comments. Now, we have added the line number. Please see the revised manuscript. |
| 2. The common previously used methods such as random forest and neural network still cannot handle missing values. Please provide supporting literature for this statement. | 2. We have rewritten the sentence and added some references (Please see the line numbers 87-89 in the Introduction part of the revised manuscript with track changes) |
| 3. Rice yield prediction has been widely studied all over the world. Please improve the introduction section by discussing about the various studies done over the world on rice yield forecasting. | 3. Thank you very much for your valuable instruction. We have added some literature regarding the rice yield and production forecasting. (Please see lines 63-73 in the introduction section of the revised manuscript) |
| 4. “The XGBoost model with the greatest result for time series data was developed by changing the parameters frequently.” What are the ranges of the parameters tried to get the best result? Please mention that. | 4. We have mentioned the tuning parameters according to the instruction (Please see the S3 Table in the Additional file 1) |
| 5. “We found the presence of heteroscedasticity and non-normality in the data.” Please present the results of statistical analysis done to test the heteroscedasticity and non-normality of the data. At the same time, present the analysis results after boxcox transformation. | 5. Thanks for your suggestions. We have removed the sentence as per our updated results and added the results of Box-Cox transformation in the supplement (Please see lines 222-224 and S1 Table in the Additional file 1). |
| 6. “we found an increasing linear trend for the annual rice production data from 1961 to 2020 in Bangladesh.” I suggest the authors to analyse the trend in the rice yield data using Mann-Kendall or linear trend analysis. | 6. We are a bit loss on how to adjust the comment. As our main aim of the study was to compare the ARIMA and XGBoost model and to forecast the rice production using the better performed model. Thus, this goes beyond our objective. However, we updated the discussion section (Please see lines 311-372 in the discussion section of the revised manuscript with track changes). |
| 7. Delete “To train these models, we used 90% of our data as training set and test the performance of the model using the remaining 10% of the data.” | 7. Thanks. We have already removed that from the manuscript (please see lines 329-331 in the discussion part of the revised manuscript) |
| 8. Discussion is merely summary of the study. You should compare your results with previously published literature. | 8. We have updated the discussion section (please see lines 311-372). |
| **Reviewer 2 Comments** | **Reviewers’ Responses to Questions** |
| The study “Accuracy Performance of Time Series and Machine Learning Models for Predicting Rice Production in Bangladesh: A Comparative Analysis” is interesting. The study is well organized and executed properly, however, attention should be given to the following highlighted points before resubmission.  1. The abstract is verbose and does not highlight the results. It should report results and main findings instead of being generic. | 1. Thanks to the reviewers for their valuable instructions. We have now improved the abstract of the study (Please see the line numbers from 25-44). |
| 2. The authors may provide some more detailed information regarding the XGBoost model which will be helpful for readers. | 2. Thank you very much for your suggestions. We have added more information on XGBoost model (please see lines 153-174 in the materials and methods section of the revised manuscript with track changes) |
| 3. The authors mentioned that they used auto.arima function for selecting the best ARIMA model. “The ARIMA models were built with the 'forecast' package using auto.arima function for choosing the best model based on the AICc values [34]”. while the authors also mentioned this statement as well. “We performed the ADF test to see the stationarity of the data and found the data non stationary (p>0.01). To compensate for the trend shift observed in (Fig 3), we used first-order differencing of the sequence (Fig 4). The differenced time series was found stationary using the ADF test (p<0.01). So, the parameter of the ARIMA model d was 1”. In the ACF diagram, there was an evident peak at lag 1 indicating that the MA may become 1 and an evident spike at lags 0 in the PACF diagram, suggesting that the AR may become 0 (Fig 5). The authors may clearly state which procedure they used to choose the best ARIMA model. | 3. Many thanks for your comments. The auto.arima function was used to list all possible ARIMA models. We have updated the text (please see lines 199-200 and 246-249 in the revised manuscript). |
| 4. What are the reasons that the authors may choose the ARIMA model with drift? This means defining the characteristics of the data. | 4. Thanks for the comment. We have updated the text (please see lines 246-249 in the revised manuscript with track changes). |
| 5. Replace Table 1 and provide the P-values of the parameters and also the complete statistics. Secondly, the authors may also update the information for XGBoost model. The tuning parameters, etc. | 5. Thanks for your valuable instruction. We replaced the Table 1 in the main text with the complete statistics. Also, we have updated the information of the XGBoost model and its tuning parameters (Please see lines 250-251, 153-174 and S3 Table in the Additional file 1). |
| 6. “We used 8 time-lagged variables as input features for XGBoost; hence, the remaining 46 values were compared for the XGBoost model”. How the 8-time lagged is selected for XGBoost. | 6. Many thanks for your feedback. We have added the explanation (please see lines 262-269 and S8 Fig.tif) |
| 7. In Table 2 I feel some doubt about reporting the results. For the testing set, the results are consistent for all accuracy criteria. While for the training set the MAE value for XGBoost is very high. In the majority of cases, the MAE value is less than the RMSE value. Please check and rectify. | 7. We have made correction in the S2 Table (Please see S2 Table in the in the Additional file 1) |
| 8. In the goodness of fit criteria, the authors may also include the directional statistics (DS) and Diebold Marino test (DM). Secondly, the MAPE results can be explained within its theoretical bounds. The authors may take help from the following studies. https://doi.org/10.1155/2020/1325071 and 10.1109/ACCESS.2019.2946992 | 8. Thanks for your suggestion. The directional statistics (DS) and Diebold Marino test (DM) in the goodness of fit criteria are more appropriate for business purposes. However, we have made correction in our error metrics (Please see line 176-194, Table 2 in the revised manuscript with track changes). |
| **Journal Requirements** | **Authors’ Responses** |
| 1. Please ensure that your manuscript meets PLOS ONE's style requirements, including those for file naming. | 1. Thank you very much to the Editors. Now, we have used the PLOS ONE’s formatting style. |
| 2. We noticed you have some minor occurrence of overlapping text with the following previous publication(s), which needs to be addressed:   Alim M, Ye GH, Guan P, Huang DS, Zhou BS, Wu W. Comparison of ARIMA model and XGBoost model for prediction of human brucellosis in mainland China: a time-series study. BMJ Open. 2020 Dec 7;10(12):e039676. doi: 10.1136/bmjopen-2020-039676. PMID: 33293308; PMCID: PMC7722837.  Rahman MS, Chowdhury AH, Amrin M (2022) Accuracy comparison of ARIMA and XGBoost forecasting models in predicting the incidence of COVID-19 in Bangladesh. PLOS Glob Public Health 2(5): e0000495. <https://doi.org/10.1371/journal.pgph.0000495>  In your revision ensure you cite all your sources (including your own works), and quote or rephrase any duplicated text outside the methods section. Further consideration is dependent on these concerns being addressed. | 2. Thanks. We have now removed the overlapping texts and corrected it. |
| 3. We suggest you thoroughly copyedit your manuscript for language usage, spelling, and grammar. If you do not know anyone who can help you do this, you may wish to consider employing a professional scientific editing service. | 3. Thank you very much for your instructions. Now, we have used Quillbot Premium for checking language, spelling, and any kind of grammar mistake and correcting it. The corresponding author (Farhana Arefeen Mila) has annual subscription for using the Quillbot software. |
| 4. We note that you have stated that you will provide repository information for your data at acceptance. Should your manuscript be accepted for publication, we will hold it until you provide the relevant accession numbers or DOIs necessary to access your data. If you wish to make changes to your Data Availability statement, please describe these changes in your cover letter and we will update your Data Availability statement to reflect the information you provide. | 4. We have now updated it. |
